# Lessons from a tuberculosis contact investigation at a federal healthcare facility during the coronavirus disease 2019 (COVID-19) pandemic

**DOI:** 10.1017/ash.2022.287

**Published:** 2022-08-26

**Authors:** Krista M. Powell, Kali Y. Crosby, Abeer A. Moanna, Alton Greene, Lauren Epstein

**Affiliations:** 1 Atlanta Veterans’ Affairs Health Care System, Decatur, Georgia; 2 Emory University School of Medicine, Department of Infectious Diseases, Atlanta, Georgia

*To the Editor* **—**The Centers for Disease Control and Prevention (CDC) has recommended universal use of well-fitting face masks or respirators in healthcare settings during the coronavirus disease 2019 (COVID-19) pandemic.^1^ This recommendation presents a unique context when estimating risk of transmission of airborne infections such as tuberculosis. In this report, we describe a tuberculosis contact investigation at a hospital-based outpatient clinic at a Veterans’ Affairs (VA) healthcare facility during the COVID-19 epidemic.

In January 2021, a healthcare provider (ie, the index case) received a diagnosis of noncavitary, miliary, 1+ acid-fast bacilli smear-positive tuberculosis disease. The index case had medication-associated immunocompromise and developed symptoms ∼1 year before diagnosis. Between September 2019 (ie, the start of the estimated infectious period^2^) and mid-March 2020 (ie, when the clinic paused in-person outpatient encounters due to the COVID-19 pandemic), the index case had >500 patient encounters. In-person encounters resumed in September 2020; by December 31, 2020, the index case had an additional 336 patient encounters in the setting of universal masking by both index case and patient. All patient encounters by the index case before and after universal masking were estimated to average 20 minutes in duration. Throughout the estimated infectious period, the index case worked in a shared office with other healthcare personnel, who ostensibly had longer cumulative exposures than patients.

Before the COVID-19 pandemic, our facility had used a 20-minute exposure threshold to initiate contact investigations for smear-positive tuberculosis cases. However, in this instance, some experts were reassured by universal masking during the latter estimated infectious period, when presumably the index case would have been most contagious. Others concluded that lack of data regarding effectiveness of universal masking to prevent *Mycobacterium tuberculosis* transmission conferred unacceptable risk, especially since annual testing was no longer used to identify transmission following the CDC’s updated recommendations for tuberculosis prevention and control in healthcare settings.^3^ Furthermore, some questioned the reliability of mask wearing because outbreaks of COVID-19 in healthcare facilities have been attributed to lack of mask wearing by healthcare personnel.^4^ Therefore, we initiated a contact investigation.

Of 46 healthcare personnel identified as contacts, 22 underwent testing for *M. tuberculosis* infection within 10 weeks of last exposure and again 10 weeks after exposure.^2^ One had a history of prevalent infection identified upon hire, but no other infections were identified, suggesting no or very limited secondary transmission. The state health department informed us that the index case’s tuberculosis genotype was unique in the state, strengthening conclusions that there was no or very limited risk of transmission. Consequently, we did not expand the investigation to include patients, a facility-level decision supported by the VA’s national review panel.

Conducting a tuberculosis contact investigation during the COVID-19 pandemic presented unique circumstances but reinforced lessons for any tuberculosis contact investigation in a healthcare setting. At the outset of the investigations, we lacked collateral public health information (eg, household contact investigation data, genotyping data) to characterize potential harm to healthcare personnel and patients. The CDC recommends that high-priority contacts, including those exposed to smear-positive tuberculosis, receive tuberculosis testing within 7 working days of identification.^2^ While seeking additional information, we proactively initiated an investigation, and we anticipated making >300 patient notifications pending findings.^5^ Fortunately, public health data provided reassurance regarding low risk of transmission, underscoring the importance of coordination of investigations with public health officials^2,5,6^ and the added value of tuberculosis genotyping data.^6,7^ As of July 11, 2022, the index case’s tuberculosis isolate’s genotype remained unique nationally. Because about half of persons with tuberculosis infection who develop tuberculosis disease do so with in the first 2 years of infection,^8^ genotyping data were even more reassuring >2 years later.

Whether to attribute the lack of transmission to index-case characteristics (ie, low-grade smear positivity, noncavitary disease, and no household transmission), the short duration of exposure and the impact of universal masking on transmission remain unclear. Although low yield at times, contact investigation remains a key component of tuberculosis elimination in the United States, facilitating identification and treatment of infected contacts and thereby averting future transmission.^9^ Healthcare facilities should utilize public health expertise and data when making tuberculosis contact-investigation decisions, especially considering lack of annual testing data for healthcare personnel and exclusive availability at the public health level of impactful data such as results of household contact investigations and genotyping data.
